# Microbial dynamics, chemical profile, and bioactive potential of diverse Egyptian marine environments from archaeological wood to soda lake

**DOI:** 10.1038/s41598-024-70411-9

**Published:** 2024-09-09

**Authors:** Ghada E. Hegazy, Madelyn N. Moawad, Sarah Samir Othman, Nadia A. Soliman, Abdelwahab Abeer E, Hussein Oraby, Yasser R. Abdel–Fattah

**Affiliations:** 1 National Institute of Oceanography & Fisheries, NIOF–Egypt, Alexandria, Egypt; 2 Pharmaceutical Bioproducts Research Department, Genetic Engineering & Biotechnology Research Institute (GEBRI), City of Scientific Research & Technological Applications, Alexandria, Egypt; 3 Bioprocess Development Department, Genetic Engineering & Biotechnology Research Institute (GEBRI), City of Scientific Research & Technological Applications, Alexandria, Egypt; 4 Medical Biotechnology Department, Genetic Engineering & Biotechnology Research Institute (GEBRI), City of Scientific Research &Technological Applications, Alexandria, Egypt; 5 Department of Chemical Engineering, Military Technical College, Cairo, Egypt

**Keywords:** Halophilic archaea, Anti–oxidant, Wadi–El–Natrun, Archaeological wood, Fossils, Microbiology, Archaea, Archaeal biology

## Abstract

Halophilic archaea are a unique group of microorganisms that thrive in high–salt environments, exhibiting remarkable adaptations to survive extreme conditions. Archaeological wood and El–Hamra Lake serve as a substrate for a diverse range of microorganisms, including archaea, although the exact role of archaea in archaeological wood biodeterioration remains unclear. The morphological and chemical characterizations of archaeological wood were evaluated using FTIR, SEM, and EDX. The degradation of polysaccharides was identified in Fourier transform infrared analysis (FTIR). The degradation of wood was observed through scanning electron microscopy (SEM). The energy dispersive X–ray spectroscopy (EDX) revealed the inclusion of minerals, such as calcium, silicon, iron, and sulfur, into archaeological wood structure during burial and subsequent interaction with the surrounding environment. Archaea may also be associated with detected silica in archaeological wood since several organosilicon compounds have been found in the crude extracts of archaeal cells. Archaeal species were isolated from water and sediment samples from various sites in El–Hamra Lake and identified as *Natronococcus* sp. strain WNHS2, *Natrialba hulunbeirensis*strain WNHS14, *Natrialba chahannaoensis* strain WNHS9, and *Natronococcus occultus* strain WNHS5. Additionally, three archaeal isolates were obtained from archaeological wood samples and identified as *Natrialba chahannaoensis*strain W15, *Natrialba chahannaoensis*strain W22, and *Natrialba chahannaoensis*strain W24. These archaeal isolates exhibited haloalkaliphilic characteristics since they could thrive in environments with high salinity and alkalinity. Crude extracts of archaeal cells were analyzed for the organic compounds using gas chromatography–mass spectrometry (GC–MS). A total of 59 compounds were identified, including free saturated and unsaturated fatty acids, saturated fatty acid esters, ethyl and methyl esters of unsaturated fatty acids, glycerides, phthalic acid esters, organosiloxane, terpene, alkane, alcohol, ketone, aldehyde, ester, ether, and aromatic compounds. Several organic compounds exhibited promising biological activities. FTIR spectroscopy revealed the presence of various functional groups, such as hydroxyl, carboxylate, siloxane, trimethylsilyl, and long acyl chains in the archaeal extracts. Furthermore, the archaeal extracts exhibited antioxidant effects. This study demonstrates the potential of archaeal extracts as a valuable source of bioactive compounds with pharmaceutical and biomedical applications.

## Introduction

Archaea constitute one of the three domains of life, alongside bacteria and eukarya. They are a diverse group of microorganisms that thrive in extreme environments, including high temperatures, acidic conditions, and high salt concentrations^1^. Among the archaea, a fascinating group known as haloalkaliphilic archaea have captured the attention of scientists due to their remarkable ability to thrive in environments with high salinity and alkaline pH levels^2,3^. Haloalkaliphilic archaea are found in various habitats, such as salt lakes, soda lakes, alkaline soils, and hydrothermal vents. These environments are characterized by extreme conditions that are challenging for most other microorganisms. The term "haloalkaliphilic" refers to their adaptation to both high salt concentrations (halophiles) and alkaline pH levels (alkaliphiles)^4^. These unique microorganisms have evolved specialized mechanisms to cope with the harsh conditions of their habitats. They possess specific adaptations at the molecular, cellular, and physiological levels that enable them to maintain homeostasis and thrive in extreme environments where other microorganisms cannot survive. One of the key adaptations of haloalkaliphilic archaea is their ability to maintain osmotic balance in high–salt environments. They achieve this by accumulating compatible solutes, such as potassium ions or organic osmolytes, within their cells. This prevents water loss and protects cellular structures from the damaging effects of high salt concentrations^5,6^**.** In addition to their osmoregulatory mechanisms, haloalkaliphilic archaea have evolved unique membrane structures and transporters that function optimally at high pH levels. These adaptations allow them to maintain cellular integrity and perform essential metabolic functions under alkaline conditions. Furthermore, haloalkaliphilic archaea display a wide range of metabolic diversity^7^. They can utilize a variety of energy sources, including organic compounds, inorganic compounds, and even light energy through the process of photosynthesis. Some haloalkaliphilic archaea are capable of using unusual electron acceptors, such as arsenate or perchlorate, which are toxic to most other microorganisms. One particular area of interest is the study of haloalkaliphilic archaea isolated from archaeological wood. Wood, especially when buried in highly alkaline or saline environments, undergoes degradation due to various factors, including microbial activity^8^. The haloalkaliphilic archaea that colonize and interact with the wood play a crucial role in its degradation process. However, recent research has shed light on another aspect of their interaction with wood—specifically, the potential anti–oxidant properties exhibited by these archaea. Haloalkaliphilic archaea isolated from archaeological wood have been found to produce bioactive compounds with anti–oxidant activity, oxidative stress, caused by an imbalance between the production of reactive oxygen species (ROS) and the body's anti–oxidant defense system, is implicated in various diseases, including cancer, cardiovascular disorders, and neurodegenerative conditions^9^. Anti–oxidants are compounds that neutralize ROS and protect the body from oxidative dam age. Haloalkaliphilic archaea have been found to produce anti–oxidants that can scavenge free radicals and reduce oxidative stress^10,11^. These anti–oxidants, such as carotenoids, polyphenols, and flavonoids, possess the ability to donate electrons and quench ROS, thereby preventing cellular damage. The unique environment in which these archaea thrive, with high salt concentrations and alkaline pH, likely contributes to the production of robust anti–oxidant compounds^12^. In this study haloalkaliphilic archaea isolated from archaeological wood and El–Hamra Lake, Wadi–El–Natrun exhibit promising anti–oxidant properties. The archaeological wood samples collected from the excavation site in Ras Gharib, Egypt's Eastern Desert, offer valuable insights into the past. This site is situated approximately 200 miles southeast of Cairo, west of the Gulf of Suez, and adjacent to the Sinai Peninsula. The wood samples hold important clues about the era in which they were used and the original preservation environment. The excavation site in Ras Gharib, Egypt, exhibits unique geological characteristics as a marine source. The region is influenced by its proximity to the Gulf of Suez and the Red Sea, which have a significant impact on the site's geological features. The area is characterized by sedimentary deposits, including marine sediments such as sand, silt, and clay, which have been transported and deposited over time by coastal currents and processes. In terms of salinity, the marine influence in this area suggests that the excavation site may have varying levels of salinity. The exact salinity levels would depend on factors such as the proximity to the sea, tidal influence, and local hydrological conditions. Regarding pH, the marine environment generally tends to have a slightly alkaline to slightly basic pH range. However, the specific pH of the excavation site in Ras Gharib would depend on local factors such as the composition of the sediments, water chemistry^13^. The bioactive compounds produced by these microorganisms have the potential to serve as natural remedies for oxidative stress–related diseases. Further research is needed to identify and characterize these compounds, understand their mechanisms of action, and evaluate their therapeutic potential. Harnessing the unique capabilities of haloalkaliphilic archaea may open up new avenues for drug discovery and development, offering novel treatments for a range of oxidative stress–related disorders. Also this study aimed to investigate the degradation of archaeological wood and to compare the antioxidant properties of halophilic archaea isolated from the archaeological wood and Wadi El Natrun. By assessing the degradation process and antioxidant potential, this research contributes to understanding the role of halophilic archaea in the preservation of archaeological materials and their potential applications in antioxidant therapies.

## Materials and methods

### Archaeal cells isolation

**Fig. 1:**
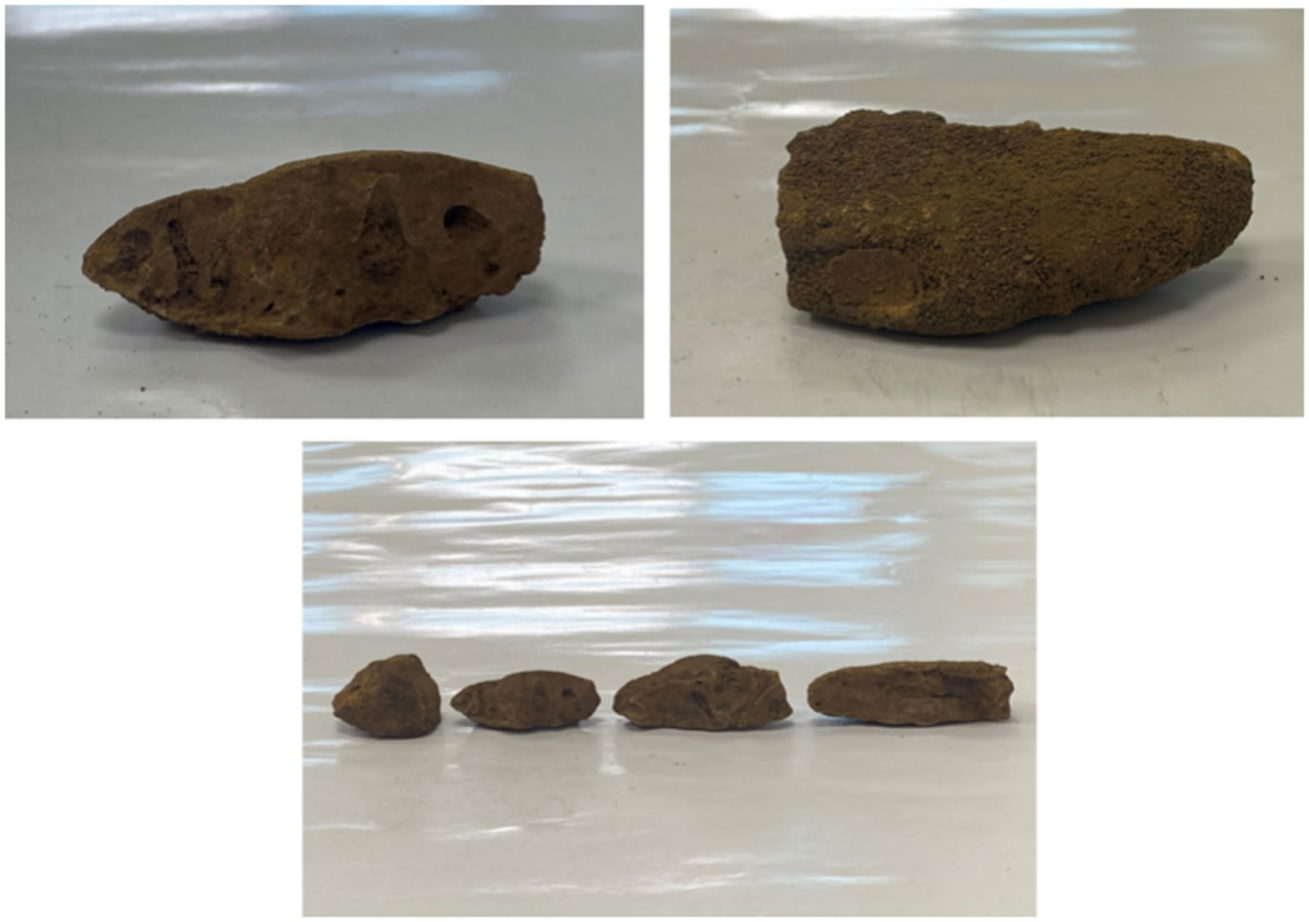
Archaeological wood samples.

**Fig. 2:**
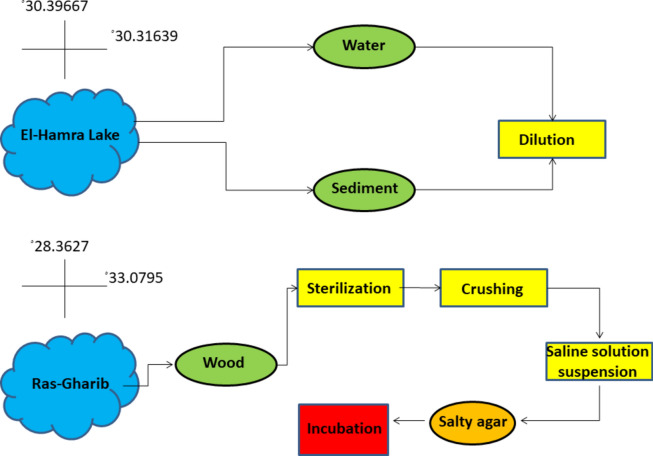
Flow chart diagram of sample collection and preparation.

Water and sediment samples were collected from various locations within El Hamra Lakes, Wadi–El–Natrun, Egypt with coordinates latitude 30.39667ﹾ and longitude 30.31639ﹾ, with pH ranging from 9.5 to 11 while salinity from 283 to 540 g/L^−1^^14^. also archaeological wood samples were collected from the excavation site in RasGharib from Egypt’s Eastern Desert site located at approximately 200 miles from southeast of Cairo and the Gulf of Suez west and the Sinai Peninsula with coordinated latitude 28.3627ﹾ and longitude 33.0795, with pH ranging from 8.8 to 9.6 and salinity range within 1000–10,000 ppm^15,16^. Water samples were serially diluted. Sediment samples were processed using a similar dilution as described for water samples. Archaeological wood samples were surface sterilized, crushed, and suspended in a saline solution. The resulting suspensions were plated on high–salt agar media with medium composition (g/L): casamino acids, 5; KH_2_PO_4_, 1; MgSO_4_·7H_2_O, 0.2; NaCl, 200; trace metals, 1 mL; and Na_2_CO_3_, 18. Trace metal solution contained (g/L) ZnSO_4_·7H_2_O, 0.1; MnCl_2_·4H_2_O, 0.03; H_3_BO_3_, 0.3; CoCl_2_·6H_2_O, 0.2; CuCl_2_·2H_2_O, 0.01; NiCl_2_·6H_2_O, 0.02; and Na_2_MoO_4_·H_2_O, 0.03 as described by Hegazy et al.^17^. Plates were incubated at the appropriate temperature for halophilic archaea growth (usually around 37 °C). The colonies showing distinct morphologies were selected and subcultured onto fresh high–salt agar plates to obtain pure cultures^17^ (Figs. 1, 2).

### Molecular identification of the selected isolates

Molecular technique, 16s*rRNA* gene sequencing, was employed to determine the phylogenetic affiliation of the isolated strains. This method depends on a rapid disruption of the selected cells from the distinct colonies picked from the surface of agar medium plates. A polymerase chain reaction (PCR) was carried out to amplify the 16s*rRNA* genes from the isolated archaeal genomes using universal primers designed to amplify ~ 1500 bp of this gene, which were then sequenced, and the BLAST program was used to assess similarity^17,18^. The 16 s rRNA partial sequences of waterlogged archaeological isolates were then deposited in GenBank under accession numbers PP177490, PP177494, and PP177495, which can be accessed via the following website https://www.ncbi.nlm.nih.gov/ by assigning these accession numbers after selecting the nucleotide.

https://www.ncbi.nlm.nih.gov/nuccore/PP177490.

https://www.ncbi.nlm.nih.gov/nuccore/PP177494.

https://www.ncbi.nlm.nih.gov/nuccore/PP177495.

Isolates from El–Hamra Lake in Wadi El–Natrun, accession numbers KP788716, KP765047, KP861849, and KP828442, are likewise similarly accessed.

https://www.ncbi.nlm.nih.gov/nuccore/KP788716.

https://www.ncbi.nlm.nih.gov/nuccore/KP765047.

https://www.ncbi.nlm.nih.gov/nuccore/KP828442.

### Archaeological wood structural evaluation

The wood structural assessment in archaeological contexts was conducted utilizing two distinct methodologies: Fourier Transform Infrared Spectroscopy (FTIR) and Scanning Electron Microscopy (SEM). Each technique necessitated specific preparatory measures to ensure optimal analysis. For the FTIR analysis, wood samples were subjected to air–drying, subsequently fragmented into small pieces, and then subjected to nitrogen purification to eliminate any residual particulate matter and wood debris. The resulting wood powder (2 mg) was mixed with potassium bromide (KBr) (200 mg) to form thin tablets under compression. Spectral analysis spanning the 4000–400 cm^−1^ region was performed using a PerkinElmer FTIR spectrometer, employing parameters of 16 scans and a resolution of 4 cm^−1^. SEM micrograph analysis entailed the utilization of a JSM IT200 scanning electron microscope equipped with a Secondary Electron Imaging (SEI) detector. Sample preparation for SEM involved dehydration via the Critical Point Dryer technique followed by gold coating. Prior to imaging, wood samples were embedded in Spurr resin and sectioned to ultra–thin dimensions (≤ 90 nm thick) using an ultra–microtome. Subsequent imaging was conducted at an accelerating voltage of 15 kV. Energy–dispersive X–ray spectroscopy (EDX) was employed for elemental analysis at selected regions of interest, utilizing a SED detector at a voltage of 20 kV and a working distance of 10 mm. Elemental composition within the SEM–observed sections was determined through this analytical approach. Additionally, elemental data was obtained through combustion analysis utilizing the ELTRA Elemental Analyzer– CHN device^19,20^.

### Preparation of archaeal crude extracts

The extraction was performed by soaking the isolated dried archaeal biomass (1: 20 w/v) in a methanol/chloroform mixture (1:1). The extract was filtered using Whatman No. 4 filter paper after being shaken for 48 h at 25 °C and 120 rpm in the dark. The pellet was repeatedly suspended in another amount of solvent. The supernatants were combined. The collected solvent was evaporated under vacuum with reduced pressure until it was completely dry, and the resulting residue, or crude extract, was then sealed in airtight bottles and kept at –20 °C until needed.

### Chemical characterization of archaeal crude extracts

#### Fourier transform infrared spectroscopy (FT–IR)

For pellets preparation, the crude extract was mashed with KBr and roughly pulverized in a mortar at a ratio of 1/100. Using a model Perkin Elmer FT–IR spectroscopy, the infrared spectra of dry extracts were examined for functional groups in the 400–4000 cm^–1^ wave number range under normal conditions.

#### Gas chromatography–mass spectrometry (GC–MS)

The chemical constituents of archaeal cell extracts were analyzed by Agilent GC–MS (Agilent model 7890A– 5975USA) equipped with an autosampler and fused–silica capillary column (DB 5MS 30 m, 0.25 mm, 0.25 μm). The injector was maintained at 200 °C using the splitless injection mode, and the injections were made at 1 μl volume. The temperature was maintained at 40 °C for 2 min then elevated to 100 °C followed by an increase to 140 °C at 2 °C/min, and finally an increase to 340 °C at 30 °C/min. The total run time was 20 min. Helium was the carrier gas at a flow rate of 0.9 ml/min. The chemical components of the archaeal cell extracts were identified (de–convoluted) using the retention indices of the GC chromatogram and the fragmentation pattern of the mass spectra by matching with the Wiley spectral library collection and the NSIT library database.

#### Total antioxidant capacity of archaeal crude extracts

The total antioxidant activity was measured using^21^ method. A green phosphate/Mo(V) complex is formed as a result of the extract's reduction of Mo(VI) to Mo(V)in acidic medium**.** Two millilitres of the reagent solution (0.6 M sulfuric acid, 28 mM sodium phosphate, and 4 mM ammonium molybdate) were mixed with a portion (0.6 ml) of the extracts. Test tubes were incubated at 95 °C in a water bath for 90 min. All samples were tested for absorbance at 695 nm against a blank after the samples had cooled to room temperature. The antioxidant capacity was given as µM ascorbic acid equivalent per gram of dry weight (µM/gdry wt).

## Results and discussion

### Chemical and structural evaluation of archaeological wood

#### Infrared analysis of archaeological wood sample

Chemical and structural evaluation of archaeological wood.

Infrared analysis of archaeological wood sample.

**Fig. 3:**
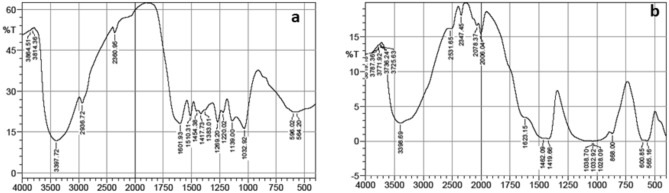
FT–IR spectra of (**a**) fresh wood and (**b**) archaeological wood.

The anoxic conditions seen in submerged environments greatly slow down degradation processes, which are mostly driven by anaerobic biological organisms. Using enzymatic breakdown, these organisms specifically target polysaccharides, with hemicelluloses being especially susceptible^22^. Since lignin has a very solid structure, it is typically thought to be far more resistant to biological deterioration. Archaeological wood is frequently marked by a high lignin concentration due to the preferred degradation of celluloses, with celluloses sometimes totally depleted^20^. While the majority of FTIR absorbance peaks are the result of several molecules, some can only be ascribed to cellulose, hemicelluloses, or lignin, as a result, they may be investigated to reveal the relative presence of each component^23^. The degree of decay is indicated by changes in the relative composition of archaeological wood as compared to fresh wood as well and the comparison of distinct peaks within the same spectrum renders the analysis semi–quantitative. The FT–IR spectra acquired from both historical and fresh samples are illustrated in Fig. 3. Within the historical specimens, notable spectral features are observed within specific wavenumber ranges. For instance, bands within the spectral region of 1000–1180 cm^–1^, indicative of stretching and asymmetric vibrations attributed to C–O, C–C, and C–O–C functionalities, alongside the band at 1375 cm^–1^ corresponding to symmetric and asymmetric bending of CH_3_ groups, are associated with the presence of cellulose and hemicellulose constituents. Additionally, a discernible reduction in the spectral intensity within the 1245 cm^–1^ region, indicative of carbohydrates and lignin, is noted. Consequently, a significant decline is observed in the 1730 cm^–1^ region, associated with the stretching of C = O bonds in xylan, a component of hemicellulose. Moreover, in higher frequency regions spanning 2800–3400 cm^–1^, attributed to C–H and OH functionalities, a substantial decrease in spectral intensity is observed, further indicating alterations in cellulose and hemicellulose content. Conversely, an increase in spectral intensity at 1505 cm^–1^, corresponding to the stretching vibrations of aromatic C = C bonds in lignin, is evident, likely attributed to the loss of extractives and carbohydrates. Table 1 illustrates the peak assignments for the FTIR spectrum of archaeological wood^24–28^.

**Table 1:** Peak assignments for FTIR spectrum of archaeological wood.

Peak position (cm^−1^)	Peak assignment	References
3700 ~ 3100 (3397.7)	Hydroxyl stretching region (O5–H5···O3 intramolecular in cellulose, O6–H6···O3 intermolecular in cellulose Iβ, and free –OH	^27,29^
2936.7	Asymmetric stretching in methylene groups	^27^
1601.9	Relative concentration of aromatic skeletal vibrations, together with the C = O stretch in the lignin	^27,29^
1510.3	C = C Aromatic skeletal vibration in the lignin	^27,29^
1454.4	C–H Deformation; asymmetric in the plane for lignin and hemicellulose	^27,29^
1417.7	CH2 scissoring in cellulose	^27,29^
1383.0	C–H bending in polysaccharides (cellulose/hemicellulose)	^27,29^
1269.2	Aromatic C–O stretching vibrations of methoxyl and phenyl propane units in guaiacol rings of lignin	^27^
1220.0	Aromatic C–O stretching vibrations in rings of lignin (C–O stretching methoxyl)	^27^
1139.0	Characteristic of the asymmetric bridge C–O–C stretching vibration in polysaccharides (cellulose/hemicellulose)	^27^
1032.9	C–O stretching vibrations in cellulose and hemicelluloses	^27^

### FTIR analysis of the archaeal extracts

The seven archaeal extracts were characterized by FT–IR spectroscopy to identify the various functional groups. The range of the FT–IR absorption peaks was predominantly between 4000 cm^−1^ and 400 cm^−1^. Different crude extracts of archaeal cells showed slight trending variations in their FT–IR absorption peaks. Table 2 gives band allocations to the respective functional groups whereas. In the current investigation, broad and strong absorption bands were found in archaeal extracts in the range of 3435.07 to 3454.44 cm^–1^. These bands were linked to the stretching vibration of the (O–H) functionality in free fatty acids, phenolic compounds, and alcohols or (N–H) group. Bands in the range of 1639.99 to 1649.7 cm^−1^ were attributed to the stretching vibration of (–C = C–) functionality of aromatic compounds and (–C = O) stretching vibration of carboxylate. The (–C–H) symmetric and asymmetric stretching vibration of long acyl chains (CH_2_ and CH_3_) appeared between 2865.32 cm^−1^ and 2965.19 cm^−1^^30^. On the other hand, the absorption bands from 1019.39 to 1095.34 cm^−1^ were attributed to the (–C–O–) functional group in alcohol and ester or to Si–O–Si in the derivatives of organosiloxanes (present in GC–MS). Moreover, the presence of Si–CH_3_ bonds is suggested by the absorption peak at about 1255 cm^−1^^31^. The bending vibrations of (–C–H) in the CH_2_ and CH_3_ groups of lipids and pigments as well as the stretching vibrations of C–N in molecules containing nitrogen (such as indole) were identified as responsible for the occurrence of vibrations at approximately 1400 cm^−1^ and 1350 cm^−1^^26^. A strong absorption peak about 650 cm^−1^ (Fig. 1S) can be observed, which suggests that a C–Cl or C–I bond is present^30^. This agrees with the chemical compounds obtained through GC–MS. These findings supported the GC–MS quantification data and further demonstrated that the crude extracts include organic silicon, ethers, organic acids, esters, and alcohols, among other substances.

**Table 2:** Bands assignment of FTIR spectra to their corresponding functional groups in different archaeal crude extracts.

Wavenumber (cm^−1^)							
Functional group	A1	A2	A3	A4	A5	A6	A7
γO–H, γN–H	3447.16	3436.09	3438.95	3440.02	3435.07	3454.44	3437.99
γ = C–H, γC–H	2959.35–2929.43	2960.42–2930.18	2965.19–2933.33	2958.69–2928.84	2956.92–2926.78–2865.32	–	2959.8–2929.66
γC═O,γC═C	1642.21	1639.99	1640.72	1641.15	1649.7	1643.07	1643.54
γC═O,γC═C	1563.62	1563.12	1564	1562.58	1564.14	1563.09	1564.5
δC–H, γC–N	1414.98–1344.86	1415.66	1414.99–1343.89	1415.65–1346.24	1461.69–1416.21–1380.69	1414.4–1344.23	1415.89–1344.74
δO–H, δSi–CH_3_	1239.23	1241.34	1241.05	1230.26	1228	1231.24	1235.84
γC–O,γSi–O	1053.41–1021.34	1051.61–1019.39	1052.19–1020.7	1053.77–1022.44	1095.34–1062.56	1053.51–1021.25	1054.28–1021.73
δC–H, δ = C–H	802.58–522.55	805.81–523.77	805.73–524.27	801.93–529.98	835.15–540.52	804.26–523.54	800.74–526.13
δC–I, δC–Cl	644.96	645.8	645.77	647.72	654.44	645.95	646.19

### Morphological feature of archaeological wood using scanning *electron* microscopy (SEM)

**Fig. 4:**
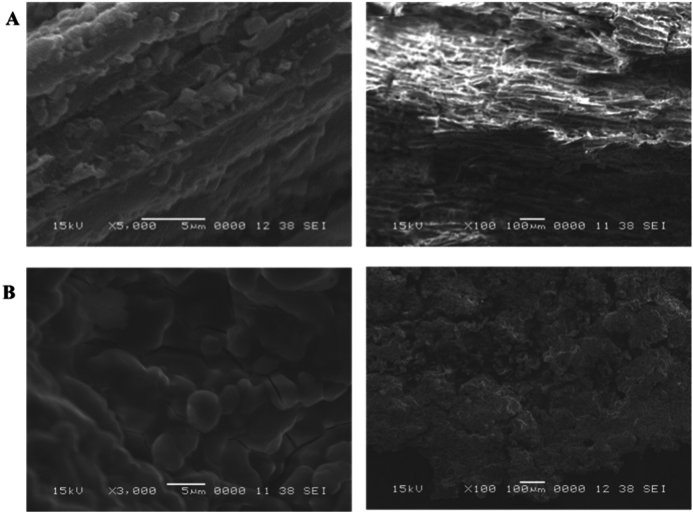
(**A**) SEM of control wood sample, (**B**) SEM of archaeological wood sample.

In wet conditions, the cellulose–depleted cell walls fill with water instead, preserving the wood's structural integrity^22^. Even though it is relatively stable, lignin may deteriorate. Nevertheless, when dried, this lignin–rich skeleton is an extremely brittle substance that is readily collapsed or warped by force^22^. As a result, the sample tended to collapse during the phases of preparation and microscopy, which had an impact on the SEM observations. The flattened and irregularly shaped wood cells in the current study are indicative of a significant degree of shrinkage upon drying, which is typical of deteriorated wood. The cell walls of archeological wood are much thinner than those of sound wood. This poor condition of preservation is confirmed by the SEM image shown in Fig. 4. This provides a clarification for the breakdown of carbohydrates detected by FT–IR measurements.

### Energy–dispersive X–ray spectroscopy (EDX) and elemental analysis of archaeological wood sample

**Fig. 5:**
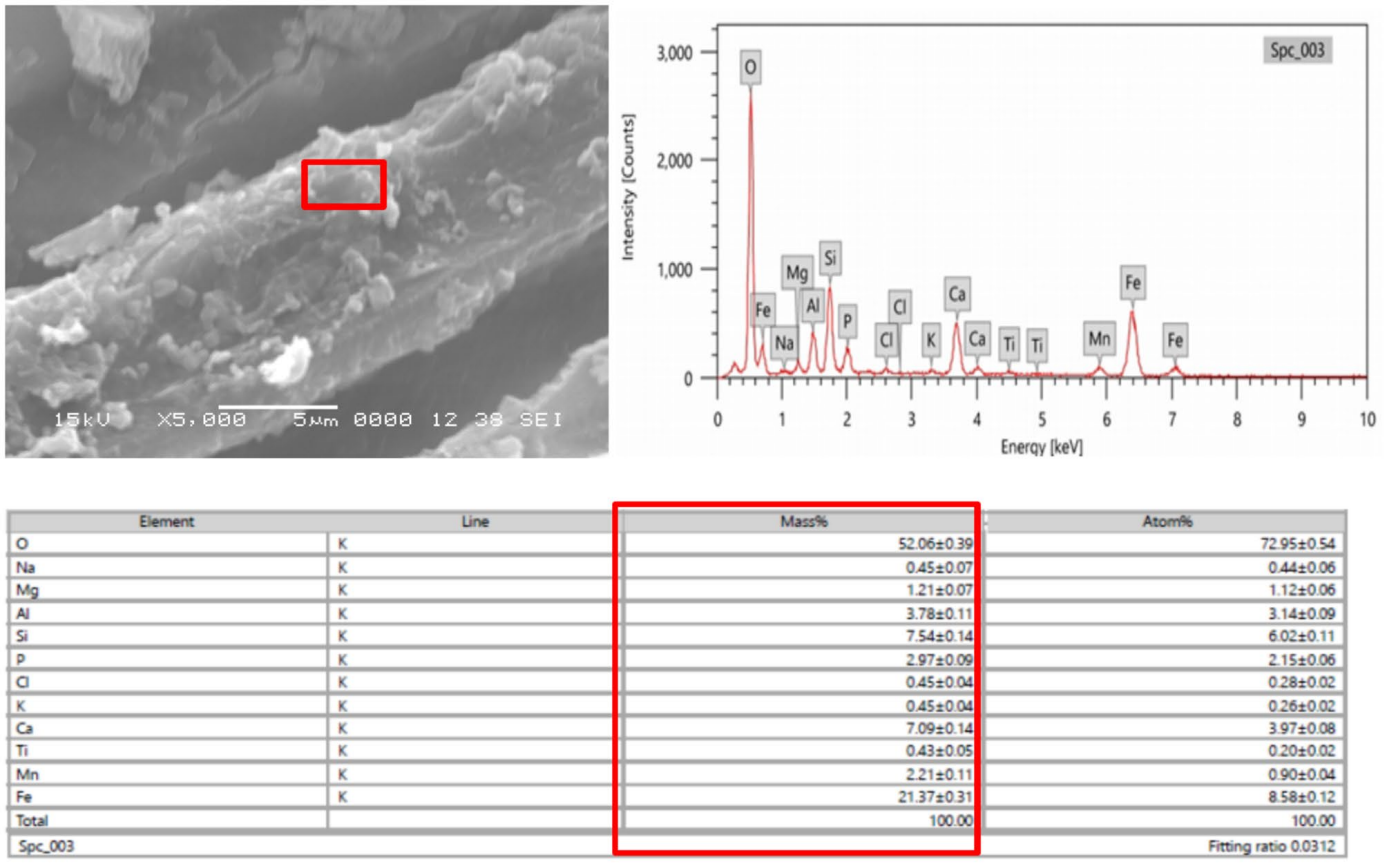
EDX analyses of inorganic deposition in archaeological wood.

During its deposition and burial, archaeological wood is exposed to a range of chemical and biological degradation processes, which produces a material that differs greatly from fresh wood in terms of its chemical composition and structure. The chemical composition of wet archaeological wood can be altered chemically as a result of exchange with the surrounding burial chamber. Since many "extractives" dissolve in water, they are either eliminated or present in much–decreased amounts. The great porosity of the wood, on the other hand, allows minerals from the burial environment, such as calcium, phosphates, and iron sulfides, to gradually seep into the cell walls, increasing the inorganic, or "ash," content. Because minerals are incorporated over time into the wood structure through interchange with the burial environment, a larger amount of inorganic components can also be a sign of decomposition^16^. SEM observation at 15 kV with a SEI detector revealed the presence of various inorganic depositions (Figs. 4,5). The presence of calcium (~ 7%), silicon (~ 7%), and iron (~ 21%) was detected by EDX analysis. Moreover, several organosilicon compounds are present in the extracts of archaea species that were isolated from archaeological samples in the current study. The literature reports that throughout the wood's burial history, the process of progressive fossilization, also known as synoptic petrification or permineralization, has occurred. This was accompanied by the buildup of minerals, including silicates (SiO_2_) and calcium carbonate (CaCO_3_)^25^. Analyses also showed the presence of oxygen (~ 52%), which is the main element in wood, and the absence of carbon. Moreover, other elements were found, including sodium (~ 0.4%), potassium (~ 0.4%), titanium (~ 0.4%), magnesium (~ 1%), phosphorus (~ 2%), manganese (~ 2%), aluminum (~ 3%), and chloride (~ 0.4%). Inorganic components can affect conservation procedures and introduce measurement errors for wood density and maximum water content, among other things. Consequently, determining the inorganic composition is a crucial step in evaluating wet archaeological wood^22^. When compared to recently submerged wood, the elemental makeup of the archaeological wood showed a lack of carbon and nitrogen, which verifies the EDX results (Table 3). It reflects the alterations and transformations undergone by the wood during the fossilization process. Archeological wood can still provide valuable information about past ecosystems and geological history.

**Table 3:** Elemental composition of archaeological wood.

Name	N%	C%	H%	S%	C/N%	C/H%
Cross section of archeological wood	0	0	0.785	0.54	0	0
Submerged wood	0.18	39.26	5.433	0.713	214.4491	7.2253

### Isolation and molecular identification of different archaeal strains

In this study, four archaeal isolates were recovered from water and sediment samples collected from different sites at El–Hamra Lake, Wadi El–Natrun and identified as *Natronococcus* sp*.* strain WNHS2 (A1) (AC, KP788716)*, Natrialba hulunbeirensis* strain WNHS14 (A2) (AC, KP765047)*, Natronococcus occultus* strain WNHS5 (A3) (AC, KP861849), *Natrialba chahannaoensis* strain WNHS9 (A4) (AC, KP828442). This lake provides a highly saline and extreme environment, making it an ideal habitat for halophilic archaea. The lake's high salt concentration and unique geochemical conditions offer a suitable niche for the survival and proliferation of these specialized halophilic archaea. In addition, three archaeal isolates from archaeological wood samples, *Natrialba chahannaoensis* strain W15 (A5) (AC, PP177495), *Natrialba chahannaoensis* strain W22 (A6) (AC, PP177494), and *Natrialba chahannaoensis* strain W24 (A7) (AC, PP177490) were selected for further investigation. All the archaea isolates were haloalkaliphilic since they could thrive in environment with high salinity and alkalinity. The biodegradation of wood, in which microorganisms break down lignin, cellulose, and hemicellulose to release carbon dioxide, water, and mineral components, is a component of the natural carbon cycle. In general, many diverse species, including fungus, bacteria, protozoa, worms, and nematodes, are involved in decomposition^24^. Hence, archaeological wood is a substrate for a wide range of microorganisms, which exist in very complex ecosystems, where interactions are possible, not only between the microorganisms, but also with other organisms. Furthermore, within the microbiota of decaying wood, microorganisms such as archaea have also been found, and clones belonging to the phyla of Thaumarchaeota, Crenarchaeota, and Euryarchaeota were identified as members of this archaeal community. However, it is still questionable whether archaea actually affects the biodeterioration of wood^32^.

### Chemical characterization of crude archaeal extracts

#### Gas chromatography–mass spectrometry analysis of archaeal crude extracts

The dried cells of seven archaeal species were extracted using solvents to produce various crude extracts to offer information that may serve as a reference for the pharmaceutical sector about the applicability of bioactive chemicals from archaeal biomass. All archaeal fractions were examined for their organic compounds using GC–MS. 59 compounds were approximately identified by GC–MS and categorized in Table (3) according to structural criteria as follows: free saturated and unsaturated fatty acids (5), saturated fatty acid esters (2), ethyl and methyl esters of unsaturated fatty acids (3), glycerides (5), phthalic acid esters (5), organosiloxane (12), terpene (3), alkane (5), alcohol (4), ketone (1), aldehyde (1), ester (11), ether (1), and aromatic (1) compounds. Numerous biological activities have been associated with the main organic compounds identified in archaeal biomass (Table 4). Cyclopropanemethanol, 2–methyl–2–(4–methyl–3–pentenyl)–, is one of the organic compounds with antibacterial and antifungal action found in crude extract of A7 isolated from archaeological wood^33^. Crude extracts of A4, A5, A6, and A7 cells have been shown to contain pentanoic acid, 5–hydroxy, 2,4–di–t–butylphenyl ester, an aromatic compound with antibacterial and anticancer activities^33,34^. Indole, another aromatic substance detected in all archaeal extracts except A1, was previously thought to be a typical contaminant in agricultural and industrial wastewater and recently was identified as a versatile signalling molecule with a broad environmental distribution. Indole has been the subject of an increasingly expanding number of studies due to its important involvement in bacterial physiology, pathogenicity, animal behaviour, and human. Numerous bacterial species have tryptophanases that may convert tryptophan to indole. Due to the diversity of environmental niches occupied by indole–producing bacteria, indole may be present in a broad range of environments, including activated sludge, soil, plant rhizospheres, the ocean, and animal and animals waste. The indole nucleus is widely employed in the pharmaceutical industry because it offers a broad range of biological actions, including anticancer, antiviral, antibacterial, anti–inflammatory, anti–HIV, and antidiabetic effects^35^. Furthermore, through interacting with the aryl hydrocarbon receptor, indole has been shown to be able to prolong health span (stay healthy and free of age–related ailments) in animals^36^, underlining the future potential of indoles as novel treatments to enhance life quality and lessen frailty. Cyclononasiloxane, octadecamethyl– was one of the polysiloxane derivatives found in the crude extract of A7 archaeal cells isolated from archaeological wood. According to reports, this substance has antioxidant[38]and antifungal properties^37^. Other polysiloxane derivatives that showed antibacterial and anticancer activities are 3–butoxy–1,1,1,7,7,7–hexamethyl–3,5,5–tris(trimethylsiloxy)tetrasiloxane[40]and hexasiloxane tetradecamethyl40], respectively. Moreover, 3–Isopropoxy–1,1,1,7,7,7–hexamethyl–3,5,5–tris(trimethylsiloxy) tetrasiloxane has been proved to be potential lead molecules for antimicrobial agent using molecular docking study^38^. Heptasiloxane, hexadecamethyl– shows activity against various target medical and agronomic pests according to^39^. Hexasiloxane, 1,1,3,3,5,5,7,7,9,9,11,11–dodecamethyl–, a siloxane derivative, functions as an emollient, solvent, antibacterial, antiseptic, and skin– and hair–conditioning agent^40^. Phthalic acid esters, a group of extensively used lipophilic compounds used as plasticizers and additives, are one of the substances that have been identified in the current study. Because of their unique chemical compositions, phthalate esters are frequently employed as synthetic materials in the production of paints, textiles, personal care products, cosmetics, and polymers. These materials have noteworthy hazardous effects^41^. The natural origin of phthalate compounds has been shown by a study on the ^14^C di–butyl phthalate level found in edible brown and green seaweeds^41^. The occurrence of phthalate esters as physiologically active secondary metabolites in plants, animals, and microbes has been noted in an increasing number of investigations^41^. Some species of freshwater algae and cyanobacteria are capable of producing di–n–butyl phthalate and mono (2–ethylhexyl) phthalate, according to^42^. Allelopathic, antibacterial, insecticidal, and other documented biological properties of PAEs may increase the competitiveness of plants, algae, and microorganisms to better withstand biotic and abiotic stress^43^. In the current investigation, phthalic acid, dibutylester (A1&A3), phthalic acid, diisooctyl ester (A2&4), phthalic acid, dodecyl nonyl ester (A5), phthalic acid, cyclobutyltridecyl ester (A6), and phthalic acid, mono(2–ethylhexyl) ester (A6&7) were found in GC–MS of different archaeal samples. The first report of phthalic acid, dodecyl nonyl ester, and cyclobutyltridecyl ester has been recorded in archaeal crude extracts isolated from archaeological wood samples. Di–n–butyl phthalate, a potent inhibitor of α–glucosidase, was isolated from *Streptomyces melanosporofaciens*, claiming by^44^. Additionally, theisolated pure phthalic acid, mono(2–ethylhexyl) ester obtained from marine–derived actinomycete Streptomyces sp. VITSJK8's shown cytotoxic action against HepG2 and MCF–7 cancer cell lines^45^. Phthalic acid diisooctylester, one of the detected PAEs, was previously isolated from the drug–producing plant *Gloriosasuperba*^46^. It can be used as an antifouling and antibacterial agent^47^. The contribution of fatty acids in crude extracts, another class of aroma compounds, was due to saturated fatty acid (n–hexadecanoic acid; octadecanoic acid; eicosanoic acid; valeric acid, 4–tridecyl ester;triacontanoic acid, methyl ester; ethyl 9–hexadecenoate) and unsaturated fatty acid (cis–vaccenic acid; oleic acid; 9–octadecenoic acid (Z)–, methyl ester; 11,14–eicosadienoic acid, methyl ester). In addition, esters of glycerol with fatty acids were found in crude extracts of archaeal cells including hexadecanoic acid, 2–hydroxy–1–(hydroxymethyl)ethyl ester;octadecanoic acid, 2,3–dihydroxypropyl ester;octadecanoic acid, 2–hydroxy–1,3–propanediyl ester; 9–octadecenoic acid, 1,2,3–propanetriyl ester; (E,E,E)–,1–Monolinoleoylglycerol trimethylsilyl ether. The pharmacological effects of this class of chemicals include antibacterial, hypolipidemic, insecticide, herbicide, anticancer, antioxidant, immunological modulators, and antispasmodic properties (Table 4). They might also be added to meals and utilised as cosmetics (Table 3). For example, Venkatramanan et al.(2020) examined the use of hexadecanoic acid, 2–hydroxy–1–(hydroxymethyl) ethyl ester in the treatment of *Chromobacterium violaceum* infections using docking score and molecular research^48^. It is obvious from the docking score and molecular dynamic investigations that this chemical binds to the CviR receptor, which suggests that it may be utilised to treat *C. violaceum* infections due to its anti–QS and antibiofilm properties. One of the significant monocyclic sesquiterpenes identified in all archaeal cell extracts except for A1is α–bisabolol, also known as levomenoli, is generated naturally from the essential oils of several edible and decorative plants. Numerous laboratory investigations have shown that bisabolol has pharmacological features that include anticancer, antinociceptive, neuroprotective, cardioprotective, and antibacterial effects. Furthermore, due to its skin–soothing properties, α–bisabolol has been utilized as a skin conditioning agent and is included in many cosmetic compositions^49^. Another volatile terpenoid identified in A2 crude extracts with antileishmanial properties is geranylgeraniol^49^. Squalene, another significant triterpene, is widely utilized in the cosmetics industry as an anti–wrinkle agent^50^ and as an anticancer, antioxidant, drug carrier, and detoxifier activities^51^ found in archaeal crude extracts except for A1 and A3.

**Table 4:** Biological activities associated with the main organic compounds identified in archaeal biomassextracts using GC–MS.

Compounds	Molecular formula	Molecular weight	RT (min)	A1	A2	A3	A4	A5	A6	A7	Class	Origin	Activity	References
Free saturated fatty acid														
n–Hexadecanoic acid	C16H32O2	256.4	15.554	+	–	+	−	−	−	−	Fatty acid	Natural product	Antibacterial Antifungal	^52,53^
Octadecanoic acid	C18H36O2	284.5	17.151–17.239	+	−	+	−	−	−	−	Fatty acid	Natural product	Antibacterial activity	^54^
Eicosanoic acid	C20H40O2	312.5	15.509–15.525	−	+	−	+	+	−	−	Fatty acid	Natural product		
Free unsaturated fatty acid														
cis–Vaccenic acid	C18H34O2	282.5	15.41	+	–	–	–	–	–	–	Trans–fatty acid (omega–7 unsaturated fatty acid)	Natural product	Antibacterial Hypolipidemic	^55^
Oleic Acid	C18H34O2	282.5	16.967–17.121	+	+	+	+	–	–	–	9–Octadecenoic unsaturated fatty acid	Natural product	Antibacterial Antifungal Insecticide Herbicide Antioxidant	^36,52,56^
Saturated fatty acid ester														
Valeric acid, 4–tridecyl ester (pentanoic acid, 4–tridecyl ester)	C18H36O2	284.5	15.523	–	–	–	–	–	–	+	Straight saturated chain of alky corboxylic acid (ester)	Natural product Unpleasant odour of stale cheese Fruity flavor	Perfumes Cosmetics Food additives Plasticizers Pharmaceuticals	
Triacontanoic acid, methyl ester	C31H62O2	466.8	15.243	–	–	+	–	–	–	–	Fatty acid methyl ester	Natural product	Antibacterial	^57^
Unsaturated fatty acid ethyl and methyl ester														
Ethyl 9–hexadecenoate (Palmitelaidic acid ethyl ester)	C18H34O2	282.5	17.192	–	+	–	–	–	–	–	Fatty acid ethyl ester	Natural product	Antioxidant Anti androgenic Flavor Hemolytic	^58^
9–Octadecenoic acid (Z)–, methyl ester	C19H36O2	296.5	16.586	+	–		–	–	–	–	Fatty acid methyl ester	Natural product		^59^
11,14–Eicosadienoic acid, methyl ester	C21H38O2	322.5	17.841	+	–		–	–	–	–	Fatty acid methyl ester			
Oleic anhydride	C36H66O3	546.9	27.626	–	–	+	–	–	–	–		NA		
Oleoyl chloride	C18H33ClO	300.9	20.72–20.729	+	–	+	–	–	–	–		Natural product		^36^
Glyceride														
Hexadecanoic acid, 2–hydroxy–1–(hydroxymethyl)ethyl ester	C19H38O4	330.5	18.099–22.444	+	–	+	–	–	–	–	Monoacylglycerols	Natural product	Pesticidal Antioxidant Antifungal Nematicide Hypocholesterolemia properties	^46,48,60,61^
Octadecanoic acid, 2,3–dihydroxypropyl ester	C21H42O4	358.6	28.491	+	–	–	–	–	–	–	Monoacylglycerols	Natural product	Food additive shows Anticancer Antimicrobial	^60^
1–Monolinoleoylglycerol trimethylsilyl ether (9–Octadecenoic acid (Z)–, 2,3–bis[(trimethylsilyl)oxy]propyl ester)	C27H56O4Si2	500.9	26.635	–	–	+	–	–	–	–	Monoacylglycerols	Natural product	Antiarthritic Anticancer Hepatoprotective Antimicrobial Antiasthma Diuretic Antioxidant Anti–inflammatory Anti–diabetic	^35,61^
Octadecanoic acid, 2–hydroxy–1,3–propanediyl ester	C_35_H_68_O_5_	568.9	18.696	–	–	+	–	–	–	–	Diacylglycerols	Natural product	Flavor	^58^
9–Octadecenoic acid, 1,2,3–propanetriyl ester, (E,E,E)–	C57H104O6	885.4	27.86	+	–	–	–	–	–	–	Triacylglycerols	Natural product	Anti–spasmodic Immune modulators	^50^
Phthalic acid ester														
Phthalic acid, dodecyl nonyl ester	C29H48O4	460.7	15.647	–	–	–	–	+	–	–	Phthalic acid ester	NA		
Phthalic acid, cyclobutyl tridecyl ester	C25H38O4	402.6	15.643–15.644	–	–	–	–	–	+	–	Phthalic acid ester	NA		
1,2–Benzenedicarboxylic acid, diisooctyl ester (phthalic acid ester)	C24H38O4	390.6	23.329–23.342	–	+	–	+	–	–	–	Phthalic acid ester	Natural product	Antimicrobial Antifouling Plasticizer	^41,47^
1,2–Benzenedicarboxylic acid, mono(2–ethylhexyl) ester (phthalic acid ester)	C16H21O4–	277.3	23.316–23.323	–	–	–	–	–	+	+	Phthalic acid ester	Natural product	Anticancer activity	^41,45^
Dibutyl phthalate	C16H22O4	278.3	15.647	+	–	+	–	–	–	–	Phthalic acid ester	Natural product	α–Glucosidase inhibitor Plasticizer	^41,44,62^
Organosiloxane														
Cyclotetrasiloxane, octamethyl–	C8H24O4Si4	296.6	6.768–6.77	–	+	–	+	+	+	+	Siloxane derivative	NA		
Pentasiloxane, dodecamethyl–	C12H36O4Si5	384.8	11.835–19.919	+	+	+	+	+	+	+	Siloxane derivative		Observed in cancer metabolism	
3–Ethoxy–1,1,1,7,7,7–hexamethyl–3,5,5–tris(trimethylsiloxy)tetrasiloxane	C17H50O7Si7	563.2	14.397–17.999	–	+	–	–	–	+	–	Siloxane derivative	NA		
3–Butoxy–1,1,1,7,7,7–hexamethyl–3,5,5–tris(trimethylsiloxy)tetrasiloxane	C19H54O7Si7	591.2	15.45–26.605	–	+	–	–	–	–	+	Siloxane derivative	Natural product	Antibacterial	^63^
Hexasiloxane, tetradecamethyl–	C14H42O5Si6	459.0	13.211–19.933	–	+	+	+	+	+	+	Siloxane derivative	Natural product	Anticancer	^52^
3–Isopropoxy–1,1,1,7,7,7–hexamethyl–3,5,5–tris(trimethylsiloxy)tetrasiloxane	C18H52O7Si7	577.2	16.574–22.669	–	+	–	–	–	+	–	Siloxane derivative	Natural product	Antimicrobial	^38^
Heptasiloxane, hexadecamethyl–	C16H48O6Si7	533.1	14.394–19.939	–	–	+	+	+	–	+	Siloxane derivative	Natural product	Against Various Target Medicaland Agronomic Pests	^39^
Trisiloxane, 1,1,1,5,5,5–hexamethyl–3,3–bis[(trimethylsilyl)oxy]–	C12H36O4Si5	384.8	11.834–22.68	+	–	+	+	+	+	+	Siloxane derivative	NA		
Cyclooctasiloxane, hexadecamethyl–	C16H48O8Si8	593.2	22.662	–	–	–	+	+	+	–	Siloxane derivative	NA		
Hexasiloxane, 1,1,3,3,5,5,7,7,9,9,11,11–dodecamethyl–	C12H36O5Si6	428.9	19.928	–	–	–	–	+	–	–	Siloxane derivative	Natural product	Antimicrobial Antiseptic Hair conditioning agent Skin– conditioning agent Emollient Solvent	^40^
Bis(pentamethylcyclotrisiloxy)hexamethyltrisiloxane	C16H48O10Si9	653.3	49.356	–	–	–	–	+	–	–	Siloxane derivative	NA		
Cyclononasiloxane, octadecamethyl–	C18H54O9Si9	667.4	22.651	–	–	–	–	–	–	+	Siloxane derivative	Natural product	Antifungal Antioxidant	^64,37^
Terpene														
alpha.–Bisabolol	C15H26O	222.4	13.691–13.695	–	+	+	+	+	+	+	Monocyclic sesquiterpenes, derived naturally from essential oils	Natural product Fruity nutty aroma	Anticancer Antinociceptive Neuroprotective Cardioprotective Antimicrobial Skin conditioning agent	^49^
Geranylgeraniol	C20H34O	290.5	31.067	–	+	–	–	–	–	–	Diterpenoid	Natural product	Antileishmanial agent	^65^
Squalene	C30H50	410.7	32.878–32.933	–	+	–	+	+	+	+	Triterpene	Natural product Faint odour	Anticancer Antioxidant Drug carrier Detoxifier Skin hydrating and emollient activities and widely used in the cosmetics industry as an anti–wrinkle agent	^50,51^
Aromatic compound														
Indole	C8H7N	117.2	10.402–10.416	–	+	+	+	+	+	+	Typical N–heterocyclic aromatic compound	Natural product	Anticancer Antiviral Antimicrobial, Anti–inflammatory, AntiHIV Antidiabetic Extend healthspan (remain healthy and free of age–related infirmities) in animals by interacting with the aryl hydrocarbon receptor	^35,36^
Alkane														
Butane, 2,2–dimethyl–	C6H14	86.2	11.967	–	–	–	–	–	+	+	Alkane	Natural product		^66^
Undecane, 3,8–dimethyl–	C13H28	184.4	14.332	–	+	–	–	–	–	–	Alkane			
Halogenated alkane														
Nonane, 1–iodo–	C9H19I	254.2	7.631–9.989	–	+	–	+	+	+	+		NA		
Decane, 1–iodo–	C10H21I	268.2	7.629–11.968	–	–	–	–	+	–	–		NA		
Hexadecane, 1,16–dichloro–	C16H32Cl2	295.3	14.967	–	+	–	–	–	–	–		NA		
Alcohol														
(2,4,6–Trimethylcyclohexyl) methanol	C10H20O	156.3	31.047	–	–	–	–	+	–	–		NA		
1–Heptanol, 2–propyl–	C10H22O	158.3	15.524	–	–	–	–	–	+	–		NA		
Threitol, 2–O–decyl–	C14H30O4	262.4	15.242–15.243	–	+	–	–	–	–	–	Higher alcohols	Natural product		^67^
Cyclopropanemethanol, 2–methyl–2–(4–methyl–3–pentenyl)–	C11H20O	168.3	31.021	–	–	–	–	–	+	–	Cyclic alcohol	Natural product	Antibacterial Antifungal	^33^
Ketone and aldehyde														
4–Heptanone, 3–methyl–	C8H16O	128.2	13.587	–	–	–	–	–	–	+		NA		
13–Tetradecenal	C14H26O	210.4	21.666	–	–	+	–	–	–		Oil	Natural product		^68^
Organic acid ester														
Oxalic acid, ethyl neopentyl ester	C9H16O4	188.2	15.24–15.246	–	–	–	+	+	+	+	Ester	Natural product	Anti–obesity activity	^69^
Oxalic acid, isohexyl neopentyl ester	C13H24O4	244.3	12.341	–	–	–	–	+	–	–	Ester	NA	Toxins	^70^
Oxalic acid, dineopentyl ester	C12H22O4	230.3	12.34	–	–	–	–	–	–	+	Ester	Natural product		
Oxalic acid, 2–ethylhexyl hexyl ester	C16H30O4	286.4	15.726	–	–	–	–	+	–	–	Ester	NA		
Malonic acid, bis(2–trimethylsilylethyl ester	C13H28O4Si2	304.5	10.28–10.284	–	+	+	+	+	+	+	Propionic acid	Natural product		^71^
Mandelic acid, di(tert–butyldimethylsilyl)–	C20H36O3Si2	380.7	16.576–19.924	–	+	+	+	+	+	+	Ester			
Mercaptoacetic acid, bis(trimethylsilyl)–	C8H20O2SSi2	236.5	13.21–18.002	–	–	+	+	–	+	+				
Benzoic acid, 2–[(trimethylsilyl)oxy]–, trimethylsilyl ester	C13H22O3Si2	282.5	8.544–8.548	–	–	–	+	+	–	–	Ester	Natural product	Benzoic acid derivatives	^72^
Propanedioic acid, (trimethylsilyl)[(trimethylsilyl)oxy]–, bis(trimethylsilyl) ester	C15H36O5Si4	408.8	14.392–19.926	–	–	+	+	––	–	–	Ester	NA		
Pentanoic acid, 5–hydroxy–, 2,4–di–t–butylphenyl esters	C19H30O3	306.4	12.203–12.208	–	–	–	+	+	+	+	Saturated fatty acid ester	Natural product	Antibacterial against foodborne bacteria Anticancer agent has β–glucuronidase	^33,34^
Benzeneethanamine, N–[(pentafluorophenyl)methylene]–.beta.,4–bis[(trimethylsilyl)oxy]–	C21H26F5NO2Si2	475.6	8.545–8.548	–	+	–	–	–	+	+				
Ether														
Trimethylsilyl catechollactate tris(trimethylsilyl) ether	C21H42O5Si4	486.9	8.549	–	–	+	–	–	–	–		Natural product		^73^

A1; 13 compounds, A2; 21 compounds, A3; 22compounds, A4; 19compounds, A5; 23compounds, A6; 22compounds, A7; 22compounds.

### Antioxidant activity

Different halophilic archaeal strains showed anti–oxidant effect. The examined strains showed antioxidant effect in which *Natrialba chahannaoensis* strain W15 was recorded the highest antioxidant activity being 90.60 µM/g followed by *Natrialba hulunbeirensis*strain WNHS14 (68.43 µM/g), *Natrialba chahannaoensis*strainW22 (17.84 µM/g), *Natronococcus* sp*.* strain WNHS2 (17.30 µM/g), *Natrialba chahannaoensis*strain WNHS9(17.18 µM/g), *Natrialba chahannaoensis* strainW24(16.65 µM/g), and *Natronococcus occults* strain WNHS5 (12.90 µM/g) crude extractsin the present study compared to control. The halophilic archaea are microorganisms that thrive in extreme environments characterized by high salt saturation, elevated temperatures, and intense UV radiation. These microorganisms have garnered interest due to the unique properties of their molecules, which exhibit remarkable tolerance to salt and heat, as well as potent antioxidant capabilities. Consequently, they serve as an excellent resource for various biotechnological applications. However, compared to other groups such as plants or algae, which are commonly recognized for their beneficial effects on health, the bioactive properties of haloarchaea have received limited attention. Therefore, Gómez–Villegas et al. (2020) presented the isolation and molecular identification of two novel haloarchaeal strains obtained from Odiel salterns in southwestern Spain. Furthermore, he investigated the antioxidant, antimicrobial, and bioactive potential of extracts derived from these strains. The findings of this research demonstrate that acetone–based extracts exhibit the highest levels of activity in anti–oxidant, anti–microbial, and anti–inflammatory assays^10^. Consequently, these extracts hold significant promise as a source of metabolites with practical applications in the fields of pharmacy, cosmetics, and the food industry. Also Ma et al., 2018 demonstrated the antioxidant activity of the carotenoids which extracted from haloarchaeon *Halorubrum* sp. HRM–150 isolated from brine water^35^. Also, Hou & Cui, 2018 indicated the anti–inflammatory, anti–oxidant, and anticancer activity of carotenoids extracts from 7 haloarchaea strains *Haloferax volcanii, Halogranum rubrum, Halopelagius inordinatus, Haloplanus vescus, Haladaptatus litoreus, Halogeometricum limi,* and *Halogeometricum rufum.* In the context of aqueous protein–rich extracts, there is currently a lack of existing reports that can be used for comparison specifically pertaining to haloarchaea^11^. The observation that various extracts derived from the studied archaea exhibit antioxidant activity suggests the presence of diverse antioxidant compounds with varying characteristics and polarities in haloarchaea. It is common for polyphenolic compounds to be associated with the antioxidant activity found in extracts from brown algae^74,75^. To date, there is no supporting evidence regarding the production of polyphenolic compounds in haloarchaea, which are typically associated with their antioxidant potential. Instead, the antioxidant capabilities of haloarchaea are primarily attributed to the presence of carotenoid pigments^76^. Nevertheless, when considering all the results collectively, they suggest that extracts from haloarchaea hold great potential as a source of antioxidant compounds that can be applied in various fields such as natural food preservation, coloration, and supplementation^77^, as well as sources of cosmetic and pharmaceutical formulations to avoid cell oxidative damage^78^ (Table 5).

**Table 5:** Total antioxidant capacity of archaeal crude extracts.

The archaeal crude extracts	Antioxidant activity (µM ascorbic acid equivalent/g
A1	17.30
A2	68.43
A3	12.90
A4	17.18
A5	90.60
A6	17.84
A7	16.65

## Conclusion

All isolated archaea were haloalkaliphilic since they could thrive in environment with high salinity and alkalinity. In this study, four archaeal isolates were recovered from water and sediment samples collected from different sites at El–Hamra Lake, Wadi El–Natrun and identified as *Natronococcus* sp*.* strain WNHS2 (AC, KP788716)*, Natrialba hulunbeirensis* strain WNHS14 (AC, KP765047)*, Natrialba chahannaoensis*strain WNHS 9 (AC, KP828442) and *Natronococcus occultus* strain WNHS5 (AC, KP861849). In addition, threearchaeal isolates, *Natrialba chahannaoensis* strain W15 (AC, PP177495), *Natrialba chahannaoensis* strain W22 (AC, PP177494), and *Natrialba chahannaoensis*strain W24 (AC, PP177490) were identifiedfrom archaeological wood samples. The presence of archaea within the microbiota of decaying wood has been reported, but their exact role in wood biodeterioration is still not fully understood. The chemical and morphological characterizations of archaeological wood were evaluated using FTIR, SEM, EDX, and combustion analysis. The FTIR analysis shows that hemicellulose, the main component of carbohydrates, degrades more than lignin. Microscopic observations of archaeological wood using SEM reveal significant changes in cell structure and morphology compared to sound wood. The presence of inorganic components, such as calcium, silicon, iron, and sulfur, has been detected through EDX and combustion analyses. Exchange with the burial environment results in the incorporation of minerals into the wood structure, increasing the inorganic content. Notably, diatoms have been reported to be related to the presence of silica in archeological wood. Archaea may also be associated with detected silica in archaeological wood since several organosilicon compounds have been found in the crude extracts of archaeal cells. In addition, the crude extracts obtained from dried cells of seven archaeal species have been analyzed using GC–MS and FTIR to identify bioactive compounds with potential pharmaceutical applications. A total of 59 compounds (A1; 13 compounds, A2; 21 compounds, A3; 22 compounds, A4; 19 compounds, A5; 23 compounds, A6; 22 compounds, and A7; 22 compounds) were identified and categorized into different structural groups. Several organic compounds exhibited promising biological activities, including antibacterial, antifungal, anticancer, anti–inflammatory, antiviral, and antioxidant effects. Overall, these findings provide valuable insights into the potential of bioactive compounds derived from halophilic archaeal biomass for pharmaceutical applications.

## Supplementary Information

Supplementary Figures.

## Data Availability

All datasets generated and/or analysed during the current study are available in the NCBI repository, (https://www.ncbi.nlm.nih.gov/) under accession numbers PP177490, PP177494, PP177495, KP788716, KP765047, KP861849 and KP828442). https://www.ncbi.nlm.nih.gov/nuccore/PP177490, https://www.ncbi.nlm.nih.gov/nuccore/PP177494https://www.ncbi.nlm.nih.gov/nuccore/PP177495, https://www.ncbi.nlm.nih.gov/nuccore/KP88716, https://www.ncbi.nlm.nih.gov/nuccore/KP765047, https://www.ncbi.nlm.nih.gov/nuccore/KP861849, https://www.ncbi.nlm.nih.gov/nuccore/KP828442.
