# Supplementary material for: Microbial dynamics, chemical profile, and bioactive potential of diverse Egyptian marine environments from archaeological wood to soda lake
Source: Sci Rep. 2024 Sep 9;14:20918. doi: 10.1038/s41598-024-70411-9 (PMC11385181; doi:10.1038/s41598-024-70411-9)
Supplement: Supplementary file 1 — Supplementary Figures. [file 41598_2024_70411_MOESM1_ESM.docx]

**Microbial dynamics, chemical profile, and bioactive potential of diverse Egyptian marine environments from archaeological wood to soda lake**

**A1**

**A2**

**A3**

**A4**

**A5**

**A6**

**A7**

**Fig. 1S FTIR spectra of different crude archaeal extracts**
